# Computational Selection of RNA Aptamer against Angiopoietin-2 and Experimental Evaluation

**DOI:** 10.1155/2015/658712

**Published:** 2015-03-19

**Authors:** Wen-Pin Hu, Jangam Vikram Kumar, Chun-Jen Huang, Wen-Yih Chen

**Affiliations:** ^1^Department of Biomedical Informatics, Asia University, Taichung City 41354, Taiwan; ^2^Department of Medical Laboratory Science and Biotechnology, China Medical University, Taichung City 40402, Taiwan; ^3^Graduate Institute of Biomedical Engineering, National Central University, Jhongli 32001, Taiwan; ^4^Department of Chemical and Materials Engineering, National Central University, Jhongli 32001, Taiwan

## Abstract

Angiogenesis plays a decisive role in the growth and spread of cancer and angiopoietin-2 (Ang2) is in the spotlight of studies for its unique role in modulating angiogenesis. The aim of this study was to introduce a computational simulation approach to screen aptamers with high binding ability for Ang2. We carried out computational simulations of aptamer-protein interactions by using ZDOCK and ZRANK functions in Discovery Studio 3.5 starting from the available information of aptamers generated through the systematic evolution of ligands by exponential enrichment (SELEX) in the literature. From the best of three aptamers on the basis of ZRANK scores, 189 sequences with two-point mutations were created and simulated with Ang2. Then, we used a surface plasmon resonance (SPR) biosensor to test 3 mutant sequences of high ZRANK scores along with a high and a low affinity binding sequence as reported in the literature. We found a selected RNA aptamer has a higher binding affinity and SPR response than a reported sequence with the highest affinity. This is the first study of *in silico* selection of aptamers against Ang2 by using the ZRANK scoring function, which should help to increase the efficiency of selecting aptamers with high target-binding ability.

## 1. Introduction

Angiopoietin-2 (Ang2) is an important regulator of vascular stability, where it has been reported that Ang2 expresses only at sites of angiogenesis, for instance, the ovary [1] and vascularized tumors [2]. Angiogenesis is the growth of blood vessels from the existing blood vessels, which is a complex process regulated by growth factors, enzymes, and extracellular matrix molecules. Of these, the tyrosine kinase receptor Tie2 is thought to be an important molecule, and its ligands are angiopoietins [3]. An Ang2 relative, termed angiopoietin-1 (Ang1), is an activating ligand that can induce the phosphorylation of Tie2, and it has the ability to facilitate endothelial cell survival and vascular impermeability. Many researchers studied the use of genetic or pharmacological targeting of Ang2 to reduce tumor angiogenesis and to delay the growth of tumors with varying degrees of effectiveness [4–6]. Many findings point out the potential therapeutic value of specific Ang2 inhibitors [7, 8], and some of the Ang2 inhibitors are already in clinical trials.

Aptamers are short single-stranded nucleic acid ligands (DNA or RNA) and are usually generated by using an* in vitro* process referred to as SELEX (exponential evolution of ligands by systematic enrichment). The SELEX procedure starts from a large library (DNA or RNA library) containing 10^14^ ~ 10^15^ different sequences, and the polymerase chain reaction (PCR) is used in the iterative cycles of selection. This selection and amplification process is usually repeated 8–12 rounds until the aptamers with the highest affinity for the target molecule are obtained. Many aptamer-based biosensors with high detection sensitivity have been constructed [9–12]. For therapeutic applications, Ang2-specific RNA aptamers have been successfully used to inhibit rat corneal angiogenesis [13] and tumor angiogenesis and growth* in vivo *[14].

Except for using the SELEX technique, Chushak and Stone proposed a computational approach to create the initial pool of RNA sequences with potential binding affinity to an anticipated target molecule for significantly accelerating the experimental screening and selection of aptamers [15]. Bini et al. [16] generated a library of 994 mutant DNA sequences based on the sequence of a well-known and characterized 15-mer thrombin-binding aptamer (TBA) for the* in silico* selection. In their study, a higher affinity binding aptamer with thrombin was screened and the simulation results were consistent with the experimental findings.

ZDOCK and the reranked approach based on the percentage of these residues/nucleotides found in the interface of each pose were used to study protein-DNA interactions [17, 18]. In this study, we used ZDOCK and ZRANK algorithms to compare the binding affinity of mutant sequences for Ang2 with that of Ang2-specific RNA aptamers according to the ZRANK score. The mutant RNA sequences were generated starting from the sequences of Ang2 aptamers published in the previous literatures [13, 14]. We tried to select mutant sequences with a higher Ang2-binding affinity than a previously known sequence with the highest binding affinity. For proving prediction accuracy, the binding experiments were carried out using a surface plasmon resonance (SPR) biosensor. We believe the computational methods proposed here are suitable for screening aptamers and also beneficial for the development of an aptamer-based biosensor.

## 2. Materials and Methods

### 2.1. Computational Assay

Fifteen Ang2 aptamers were collected from various literatures [13, 14], and the Ang1 specific RNA aptamer (named as Seq16 in this study) [19] was considered to be used as the control group in the SPR experiment, as Seq16 is an experimentally proved Ang1 specific aptamer and should have the worst binding affinity than other 15 aptamers.  Table 1 shows the truncate and original 16 sequences of RNA aptamers collected from previous studies. Previous study demonstrated that Seq1 had a stem-loop structure and still could bind to Ang2 with a *K*_*d*_ of 2.2 nM (95% CI 1.3–3.8 nM), similar to the full-length aptamer; even it was a 41 nt truncate of aptamer [13]. Besides, the aptamer named as Seq15 in this study could partially inhibit Tie2 phosphorylation by an equimolar concentration (15 nM) of aptamer in the pegylated form and is completely blocked by a 10-fold molar excess [14]. For generating 3D structures of aptamers, these sequences were initially converted to 2D structures with the help of a web server: CentroidFold (http://www.ncrna.org/centroidfold), which yields dot-bracket notation of sequences [20]. The dot-bracket notation information of each aptamer was utilized to generate three-dimensional models with help of the RNAComposer. The web server is an automated RNA structure modeling server and promises the prediction of large RNA 3D structures of high quality (http://rnacomposer.ibch.poznan.pl/Home) [21]. The RNA 3D structure of Seq15 was shown in Supplementary Material (see Figure S1 in the Supplementary Material available online at http://dx.doi.org/10.1155/2015/658712). With the aim of studying the interaction among Ang2 and its aptamers, angiopoietin-2/Tie2 complex crystal structure (code: 2GY7) was downloaded from the Protein Data Bank (PDB). The crystal structure of Ang2-Tie2 complex was produced from the study by Barton et al. [22] and the resolution of this complex is 3.7 Å. The software for molecular simulation used in this study was Discovery Studio 3.5 (DS 3.5; Accelrys Inc., San Diego, USA). We used DS 3.5 to read the PDB file and removed the molecular structure of Tie2 in order to obtain the structure of Ang2. The isolated structure of Ang2 contains 216 amino acids, and an intact structure of Ang2 should have 496 amino acids. However, the isolated structure of Ang2 already contains the interacting domain of Tie2. The structure of Ang2 was used in the following molecular simulations. All the computational experiments/simulations were performed on a workstation with a 3.20 GHz Intel Xeon E3-1230 processor and 4 GB RAM supported by Windows Server 2008 R2. The computational simulation of each aptamer-Ang2 complex took around 15 to 35 hours.

**Figure 1:**
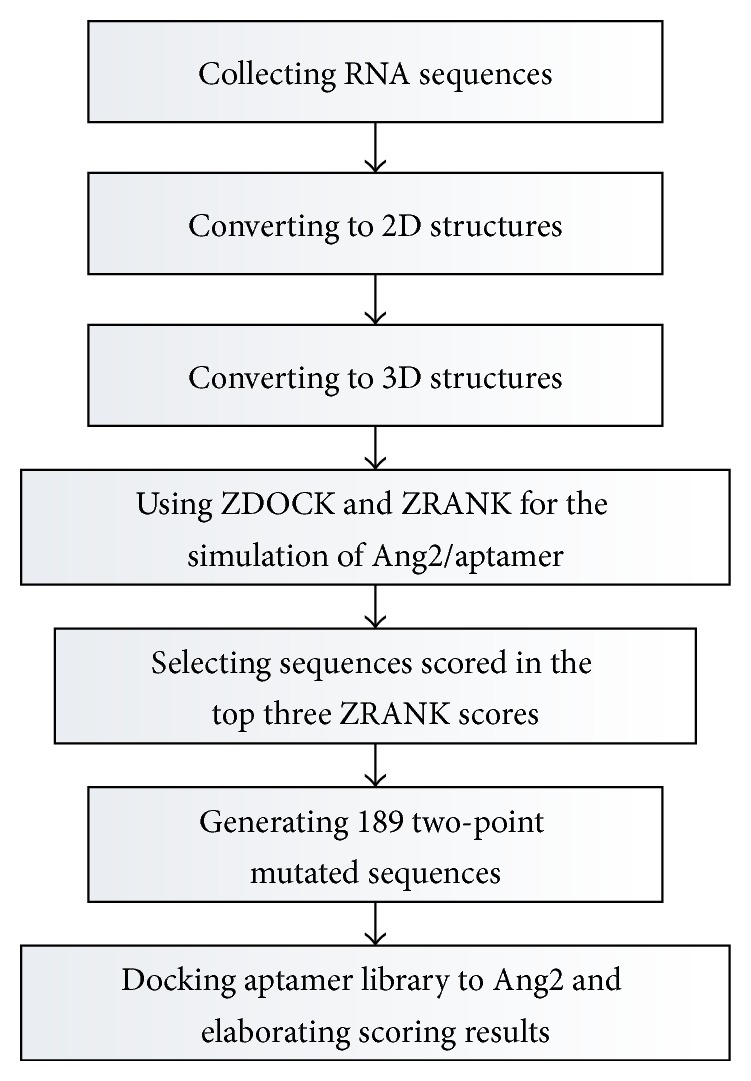
Flow chart of computational simulations.

The ZDOCK algorithm in Discovery Studio uses the fast Fourier transform (FFT) correlation techniques and searches all possible binding positions of the two proteins. The original scoring function of ZDOCK is a geometrical measure according to the degree of shape complementarity between the two docking proteins. For the software package DS 3.5, the ZDOCK program incorporates the pairwise shape complementarity scoring function for identifying docked conformations and scores hits based on atomic contact energies. In the past study, the degree of shape complementarity (SC) was used to quantify the interaction between two interacting surfaces [23], and the thrombin-aptamer complex had better SC than most antibody/antigen interactions [24, 25]. Based on the calculation of shape complementarity, we used the ZDOCK algorithm to study the aptamer-Ang2 interaction. In addition, the ZRANK scoring function was also applied to each simulation in this study that reranked initial-stage ZDOCK predictions with detailed electrostatics, van der Waals, and desolvation energy terms. In our previous research, we adopted the ZDOCK method to simulate the interactions between the thrombin and aptamers, and simulation results were consistent with experimental results from literatures [26].  Figure 1 illustrates the main computational procedure steps in the simulations.

### 2.2. The Library of Mutant RNA Sequences

The DS 3.5 software provides a function to explore the binding interface of molecular interaction. From the analysis of all binding interface between Ang2/aptamers, it was found in common that most of the nucleotides in RNA aptamers from positions 10 to 16 and approximately to the end of the sequence are actively involved in binding with Ang2 (data shown in Supplemental Table S1). Therefore, we decided to produce mutant sequences by replacing the nucleotides in the original sequence at positions 10 to 16 and 33 to 39. Take Seq1 as an example, in which the mutant sequences were produced by the subsequent description rules.

Seq1 (5′-ACUAGCCUC**A**UCAGCUCAUGUGCCCCUCCGCC**U**GGAUCAC-3′) is mutated by replacing the nucleotide “A” at position 10 with “U” and changing “U” at position 33 to “A.” All possible 2-point mutations executed at positions 10 and 33 are listed as follows:

 (A10U and U33A): 5′-ACUAGCCUC**U**UCAGCUCAUGUGCCCCUCCGCC**A**GGAUCAC-3′;
 (A10G and U33G): 5′-ACUAGCCUC**G**UCAGCUCAUGUGCCCCUCCGCC**G**GGAUCAC-3′;
 (A10C and U33C): 5′-ACUAGCCUC**C**UCAGCUCAUGUGCCCCUCCGCC**C**GGAUCAC-3′;
 (A10U and U33G): 5′-ACUAGCCUC**U**UCAGCUCAUGUGCCCCUCCGCC**G**GGAUCAC-3′;
 (A10G and U33C): 5′-ACUAGCCUC**G**UCAGCUCAUGUGCCCCUCCGCC**C**GGAUCAC-3′;
 (A10C and U33G): 5′-ACUAGCCUC**C**UCAGCUCAUGUGCCCCUCCGCC**G**GGAUCAC-3′;
 (A10U and U33C): 5′-ACUAGCCUC**U**UCAGCUCAUGUGCCCCUCCGCC**C**GGAUCAC-3′;
 (A10G and U33A): 5′-ACUAGCCUC**G**UCAGCUCAUGUGCCCCUCCGCC**A**GGAUCAC-3′;
 (A10C and U33A): 5′-ACUAGCCUC**C**UCAGCUCAUGUGCCCCUCCGCC**A**GGAUCAC-3′.

Likewise, mutations were done at positions 11 and 34 for Seq1; and this procedure was progressively followed until reaching positions 16 and 39. From each aptamer sequence, 63 mutant sequences were able to be generated. Therefore, the library contained a total of 189 mutant sequences that were created from the sequences with the top three ZRANK scores (Seq1, Seq2, and Seq15). All of them were converted to 3-dimensional structures to calculate the binding affinity to Ang2 with the ZDOCK and ZRANK algorithms.

### 2.3. Reagents and Aptamers

Poly(ethylene glycol) thiol (HS(CH_2_)_6_(EG)_3_OH) was purchased from ProChimia (ProChimia Surfaces Sp. z o.o., Sopot, Poland), and biotinylated alkanethiol (BAT) (C_31_H_58_N_4_O_6_S_2_) was purchased from SensoPath Technologies (SensoPath technologies Inc., Bozeman, USA). Streptavidin was bought from Merck (Merck KGaA, Darmstadt, Germany). Tris buffered saline (TBS) (1X solution, pH 7.4) was acquired from ECHO Chemical Co., Ltd. (ECHO Chemical, Miaoli County, Taiwan). Active human angiopoietin-2 was purchased from Abcam (Abcam plc., Cambridge, UK). All other chemicals used in this study were reagent grade. The selected RNA sequences of aptamers used in this study are listed in Table 2. The RNA sequences were named based on the original sequence name and joined with the positions of mutant nucleotides in the original sequences. All of the aptamers were synthesized and purchased from Geneisland (Geneisland Corp., New Taipei City, Taiwan). For immobilizing these aptamers on the sensor surfaces, each sequence was modified with a biotin at the 5′ end and five consequent adenines were used as the spacer for yielding well steric accessibility of the aptamer.

### 2.4. Experimental Assay

**Figure 2:**
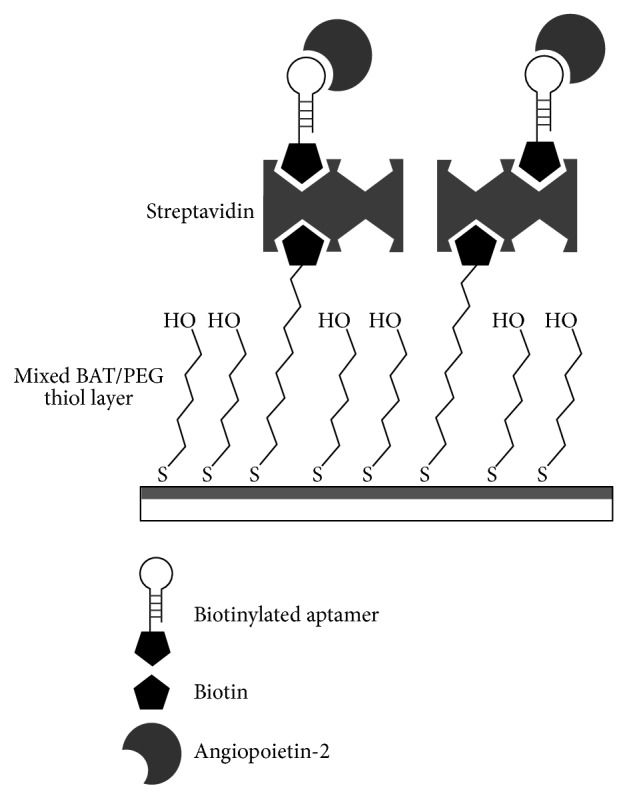
The scheme of the sensor chip. Schematic representation of mixed BAT/PEG thiol layer, streptavidin, and biotinylated RNA aptamers on the SPR sensor surface.

The SPR sensor platform we used in this study was an SPR imaging system with six flow chambers, which was developed by the Institute of Photonics and Electronics (IPE, Prague, Czech Republic) [25, 26] and as briefly described in the Supplementary Material. Before performing SPR experiments, the sensor chips were rinsed with abundant deionized (DI) water and absolute ethanol before being blown dry with nitrogen. The chips were then cleaned by an ultraviolet- (UV-) ozone treatment for 20 min, followed by washing thoroughly with DI water and absolute ethanol. The chips were blown dry with nitrogen again. The clean chips were subsequently immersed in a 1 mM mixed BAT/PEG thiol solution (1 : 9 molar ratio) for 16 h. Finally, the chips were rinsed with DI water and then blown dry with nitrogen. After generating the BAT/PEG patterns, the SPR chips were ready to be installed in the instrument. The surface of the SPR chip was then functionalized by sequential flow of a TBS solution containing 1 *μ*M streptavidin and a solution consisting of the TBS and 0.5 *μ*M biotinylated RNA aptamers. The streptavidin molecules are expected to bind specificity with the biotin moieties on the BAT/PEG patterns. A 15 min injection of TBS solution containing 1 *μ*M streptavidin was performed for depositing streptavidin molecules on the sensor surface, followed by a TBS rinse. Each streptavidin molecule can provide four biotin binding sites, and the streptavidin layer was then ready to bind with the biotinylated RNA aptamers. A solution with biotinylated RNA aptamers flowed over the patterned BAT/PEG layer for 15 minutes and was then followed by a TBS wash. After the TBS wash, the TBS solution containing 0.5 *μ*M angiopoietin-2 was flowed into the flow channels in the SPR instrument for 9 min, followed by a TBS rinse for 6 min. A schematic representation of the mixed BAT/PEG thiol layer, streptavidin, and biotinylated RNA aptamers on the SPR sensor surface is shown in Figure 2. All experiments were performed in duplicate and data were collected from six defined sensing areas in each channel of SPR imaging apparatus. The SPR experimental data were fitted by two mathematical equations using Matlab 2013b (The MathWorks, Inc., USA) for the determination of the association rate constant *k*_*a*_ and the dissociation rate constant *k*_*d*_ in the first-order kinetics model [27]. These equations are listed as follows:

(1) $$\begin{array}{c} R_{t}=R_{0}e^{-k_{d}(t-t_{0})}, \end{array}$$

where *R*_*t*_ is the SPR signal at time *t* and *R*_0_ is the value of the initial SPR signal at time *t*_0_. The association rate constant was derived by using the following equation:

(2) $$\begin{array}{c} R_{t}=\frac{\left(1-e^{-(k_{a}C+k_{d})t}\right)k_{a}CR_{\max}}{k_{a}C+k_{d}}, \end{array}$$

where *R*_max⁡_ is the largest SPR signal corresponding to the maximum analyte binding capacity and *C* is the concentration of the injected analyte. The binding affinity, *K*_*A*_, is defined in accordance with the relationship *K*_*A*_ = *k*_*a*_/*k*_*d*_.

## 3. Results and Discussion

### 3.1. Selection of Aptamers from Computational Results

In our previous report, the ZDOCK scoring function had the capability of giving accurate docking results for the interactions between thrombin and 15-mer aptamers [26]. For evaluating the feasibility and rationality of using ZDOCK and ZRANK algorithms, we took the 16 RNA aptamers with known characteristics to perform the simulations in the initial stage of this study. Then, we found that the ZRANK score could give more trustworthy results, which were consistent with the experimental results in the literatures (shown in Table 1). For example, the Ang1 specific aptamer (Seq16) got the worst score, and the aptamer bound Ang2 with highest affinity (Seq1) had the best score. Therefore, we decided to use the ZRANK score for evaluating the interactions between aptamers and Ang2. We think the reason for the inaccurate predictions of RNA-protein interactions by using the ZDOCK scoring function is that the RNA aptamers used in this study have longer lengths and more complex 3D structures. Hence, a more detailed energetic based scoring function known as ZRANK was needed for reranking the docked protein poses with the purpose of improving the success rate of the ZDOCK predictions. The ZRANK scoring function takes repulsive energies, van der Waals attractive, electrostatic short and long range attractive, and repulsive energies into calculations.

For the sequences listed in Table 1, the top three ZRANK scores were −93.855, −82.722, and −80.325 for Seq1, Seq2, and Seq15, respectively. Besides, Seq16 obtained the lowest ZRANK score of −61.969. Basically, these docking results are consistent with the previous experimental studies [13, 14, 19]. Seq1 can bind Ang2 with high affinity (*K*_*d*_ of 2.2 nM) [13], and Seq16 does not have specificity for Ang2 (*K*_*d*_ > 1 *μ*M) [19]. A number of 189 mutant RNA aptamer sequences were generated from three reference structures of aptamers, and all results were provided in Supplementary Material (Table S2). After simulations, three mutant aptamers, Seq2 mutated at position “12” and position “35,” Seq15 mutated at position “12” and position “35,” and Seq15 mutated at “15” and “38,” showed high binding affinity (according to the ZRANK scores) to Ang2 compared with their original aptamers. These three mutant aptamers were taken into consideration to test their binding affinity in experimental conditions. Besides, we also analyzed the binding interface of aptamer-protein complex in the best pose of each simulation.

**Figure 3:**
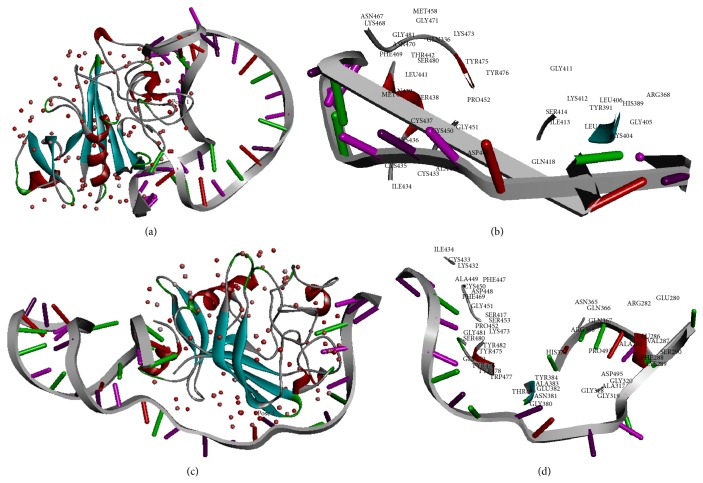
Analysis of docking results. The docking predictions of aptamer-protein complexes and the amino acid residues involved in the binding interfaces. The locations of red dots indicate the center of the binding interface on the receptor structure for each pose. Amino acids in the Ang2 involved in the binding interaction with each aptamer are detailed, marked with amino acid position numbers. (a) Complex of Ang2/Seq15_12_35. (b) Binding interface between Ang2 and Seq15_12_35. (c) Complex of Ang2/Seq15_15_38. (d) Binding interface between Ang2 and Seq15_15_38.

By exploring the binding interface of molecular interaction, the amino acids in the binding interface between Ang2 and an aptamer can be analyzed for finding out whether the aptamer with the best pose binds with the Ang2 receptor-binding domain or not. K468, F469, K473, Y475, and Y476 are five important residues in Ang2 that constitutes the binding domain for Tie2 [22, 28]. Taking the binding site of Ang2 into consideration, Y475 and Y476 in Ang2 participated in the binding reaction for the best docked pose of Seq1 in our study. As for Seq2_12_35, Y475 and Y476 in Ang2 participated at the interface site of the best docked pose.  Figure 3 illustrates the best poses of Seq15_12_35 and Seq15_15_38 for docking with the Ang2 and shows amino acids involved in the binding interactions. Five critical residues in Ang2 for Tie2 binding were all confirmed to take part in the docking interface of Seq15_12_35 and Ang2. Considering the Seq15_15_38, there were four important residues (F469, K473, Y475, and Y476) involved in the binding interface between the aptamer-protein complexes. Y475 and Y476 were also involved in binding interaction of the Ang2/Seq16 complex. Therefore, these results demonstrated that the five amino acid residues in the interacting domain of Ang2 fully or partially interacted with the best docking poses of these aptamers.

### 3.2. Comparison on the Ang2-Binding Affinity Using SPR Experiments

**Figure 4:**
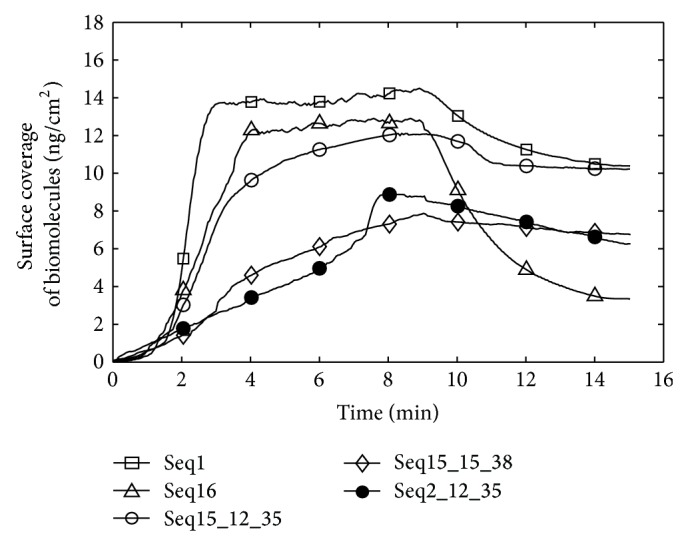
SPR sensorgrams. Representative SPR sensorgrams for interactions between immobilized Ang2 and different aptamers.

Figure 4 shows the representative SPR sensorgrams of different aptamers. The average and standard deviation values of sensor responses for different aptamers expressed by the surface coverage of biomolecules are listed in Table 3. For Seq1 and Seq16, the final experimental results were consistent with the simulation scores and well-known characteristics. The experimental results indicated that the Seq15_15_38 could generate an SPR signal that was approximate to the SPR response of Seq1 in the end of the experiment. The Seq15_15_38 had a slightly larger average experimental value compared with Seq1. Nevertheless, the ZRANK score of Seq15_15_38 was a little lower than that of Seq1. For other two mutant sequences, Seq15_12_35 and Seq2_12_35, they exhibited a slightly worse performance than Seq1, which were not in agreement with the simulation findings. Actually, a number of factors can influence the kinetics of protein-protein interactions, like viscosity, pH, and ionic strength of a solution [29]. We suggest that the ZRANK scoring function does not take the buffer conditions into the calculation, which is the major reason for the differences in the experimental and computational results.

For further understanding of the interactions between the Ang2 and five sequences, kinetic parameters were calculated. Table 3 exhibits the kinetic parameters for Ang2/aptamer interactions. The highest *k*_*a*_ value was found in the Ang2/Seq1 interaction, and Seq16 had the lowest *k*_*a*_ value among the five aptamers. The Seq16 had the largest dissociation rate constant, which means the interaction between the immobilized Ang2 and Seq16 is very weak. We think that the large SPR response for Ang2 and Seq16 in the association phase is contributed by the nonspecific adsorption of Seq16 on the sensor surface. It should be noticed that the three mutant sequences all had smaller dissociation rate constants. Regarding the strength of interaction between two molecules, we can assess it by determining the value of binding affinity, *K*_*A*_. Among these five mutant aptamers, the Seq15_12_35 had the largest *K*_*A*_ value, and Seq16 had the worst *K*_*A*_ value. This result means that Seq15_12_35 can have a tight binding reaction with the Ang2. Like the Seq15_12_35, the Seq15_15_38 also had a bigger *K*_*A*_ value than Seq1. For the mutant sequence Seq2_12_35, the mutant sequence Seq2_12_35 got the best ZRANK score in the computational simulation, but it just had the *K*_*A*_ value of 5.15 × 10^6^ M^−1^. We noticed that simulation results for Ang2-Seq1 or the Ang2-Seq16 complexes were in agreement with the experimental findings. For the three mutant sequences, our experimental findings were not fully consistent with the computational results. These results demonstrated that the ZRANK scoring function could give simulation results in accordance with the real situations when aptamers exhibited apparent differences in the interactions with Ang2. We suggest that some factors such as ionic strength and pH of buffer may influence the real interactions to cause different results than expected in simulations.

Savory et al. [30] performed post-SELEX screening using the genetic algorithm (GA) with the preselected DNA sequences to improve the prostate specific antigen- (PSA-) binding ability of aptamers. Initially, they generated 20 oligonucleotide sequences by using GA with the five sequences preselected through SELEX. For producing the third generation, they selected the top four sequences that showed higher PSA-binding ability in the second generation. Then, a random one-base mutation was introduced into the production of third and fourth generations of sequences. Nevertheless, not all mutant sequences could have a high PSA-binding ability. Finally, an oligonucleotide was found to have the highest PSA-binding ability in the fourth generation oligonucleotides, which was 48-fold higher than that of the parent oligonucleotides. A previous report indicated the truncate aptamer Seq1 possessed a high binding affinity to Ang2, similar to the full-length aptamer of 81 nt [13]. Concerning efficient chemical synthesis, aptamers with shorter lengths are more ideal for syntheses, especially for the length of sequence less than 50 nt, and truncate aptamers are much more suitable for applications. However, the Ang2-binding ability of these truncate aptamers had not been proven and disclosed in the past report for other oligonucleotide sequences from Seq2 to Seq15. Another literature showed that the pegylated Seq15 had an enhanced bioavailability and therefore would weaken angiogenesis and inhibit tumor growth* in vivo *[14]. Except for Seq16, the aptamer sequences used in our study basically already have the Ang2-binding ability, and Seq1 has the strongest binding affinity to Ang2 among these sequences. Unlike the literature reported by Savory et al., we just found two mutant sequences with slight improvements in the binding affinity by using the proposed strategy in this study. These two selected sequences with higher binding affinity to Ang2 were all generated from Seq15, and the best one was Seq15_12_35. This outcome could demonstrate that our proposed strategy was feasible to apply in the selection of aptamers for achieving the improvement of binding affinity to Ang2 by using original sequences with Ang2-binding ability.

## 4. Conclusion

In this paper, we summarize the application of ZDOCK and ZRANK algorithms which basically can be used for the* in silico *selection of aptamers and have the capacity of selecting an aptamer with a higher target-binding ability for using in the biosensors and diagnostics. For verifying the accuracy of simulation, we carried out the experiments on the SPR biosensor to study the real binding reactions between aptamers and Ang2. In this study, we identified that a selected Ang2 aptamer had a higher binding affinity and SPR response than a well-known sequence with high affinity in the literature. Indeed, computational approaches can help reduce time and money consumption and increase chances of success in the selection of aptamers with high affinity to target proteins. For validating the real performance, we think that experimental screening procedures remain indispensable for testing and validating the target-binding ability of aptamers selected from simulations.

## Supplementary Material

We used the CentroidFold and RNAComposer webservers to generate the 3D structural model of Seq15. The names and numbers of RNA nucleotides are labeled in the Figure S1. This model shows that the single-stranded RNA folds into a complex and particular shape. This shape of the single-stranded RNA is critical to the binding interaction between the aptamer and its target. Because shape complementarity, polar contacts, hydrogen bonding interactions, and charge-charge interactions are important for aptamer-target recognition.
Table S1: Nucleotides of three RNA aptamers involved in the binding interface with Ang2. Nucleotides at position 10 to 16 and 33 to 99 were marked with bold, underlined text.
Table S2: The simulation results of 189 mutant RNA aptamer sequences. The mutated positions in the sequences and the ZRNAK scores of three selected aptamers were marked with bold letters.

## Figures and Tables

**Table 1:** Sequences of RNA aptamers. Seq1~Seq15 are Ang2-specific aptamers, and Seq16 is an Ang1 specific aptamer.

Name	Sequence (5′-3′)	ZRANK score
Seq1	ACUAGCCUCAUCAGCUCAUGUGCCCCUCCGCCUGGAUCAC	−93.855
Seq2	UUAACCAUCAGCUCAUGGCCCCUGCCCUCUCAAGGACCAC	−82.722
Seq3	CACCAGACCGACAUCAGCUUAUGGCCCCUCACCCACACCG	−73.128
Seq4	CCACCGAUCGCAUCAGCUCAUGGCCCCUCCCGACCCGCCA	−72.227
Seq5	CCAGACGUUCUCGCCCCGCCGAUCAUCAGCGCUGGCCCUAU	−69.183
Seq6	CACUACCACGCCAUAUCAGCUAAUGGCCCCUCCCUACGCA	−73.305
Seq7	ACUCACCAGUCACCAUCAGCUCAUGCGCCCCUCCCCCGAC	−63.518
Seq8	UGACCAAGCCUCACGUUGAACCUGCCAGUAGACCCCGCCCA	−70.795
Seq9	GGAGCGCAAUUCGCCUCGCAAGUUGAACUCCGCUGGCGG	−74.153
Seq10	UAAGCUCUUUGGCUUAGCCCGACACGUUGAACUCCAGAGU	−65.73
Seq11	CACGGUACCACCAAGUCACACGUUGAACUCCAUGCAGCUG	−62.02
Seq12	CAUGUCUACAACAAUCUCGCCCGUUGAGUCUCGUCGAAUU	−68.159
Seq13	CACUCAGCGCCCUGCGAAACGUUGCCGCCUCCCAACGUCU	−74.432
Seq14	CUCUUUUUGUCCCCGCACGUUGAACUCCUGUCCCUCUACU	−73.895
Seq15	GAGGACGAUGCGGACUAGCCUCAUCAGCUCAUGUGCCCCUC	−80.325
Seq16	ACUCGAACAUUUCCACUAACCAACCAUACUAAAGCACCGC	−61.969

**Table 2:** The RNA sequences used in the SPR experiments.

Name	Sequence
Seq15_12_35	5′-biotin-AAAAAGAGGACGAUGC**C**GACUAGCCUCAUCAGCUCAUGU**C**CCCCUC-3′
Seq2_12_35	5′-biotin-AAAAAUUAACCAUCAG**A**UCAUGGCCCCUGCCCUCUCAAG**C**ACCAC-3′
Seq15_15_38	5′-biotin-AAAAAGAGGACGAUGCGGA**U**UAGCCUCAUCAGCUCAUGUGCC**G**CUC-3′
Seq1	5′-biotin-AAAAAACUAGCCUCAUCAGCUCAUGUGCCCCUCCGCCUGGAUCAC-3′
Seq16	5′-biotin-AAAAAACUCGAACAUUUCCACUAACCAACCAUACUAAAGCACCGC-3′

**Table 3:** Experimental data and the computationally obtained scores.

Name of aptamer	Surface coverage of biomolecules (ng/cm^2^) (AVG ± SD)	*k*_*a*_ (×10^3^ M^−1^s^−1^)	*k*_*d*_ (×10^−3^ s^−1^)	*K*_*A*_ (×10^6^ M^−1^)	ZRANK score
Seq1	11.17 ± 1.47	10.02	1.39	7.23	−93.855
Seq16	1.87 ± 0.31	1.66	4.99	0.33	−61.969
Seq15_12_35	8.12 ± 0.61	6.03	0.61	9.89	−93.335
Seq15_15_38	11.69 ± 1.11	8.22	0.97	8.47	−89.904
Seq2_12_35	5.68 ± 0.41	4.07	0.79	5.15	−97.609
