# Supplementary material for: Computational Selection of RNA Aptamer against Angiopoietin-2 and Experimental Evaluation
Source: Biomed Res Int. 2015 Mar 19;2015:658712. doi: 10.1155/2015/658712 (PMC4383501; doi:10.1155/2015/658712)
Supplement: Supplementary file 1 — We used the CentroidFold and RNAComposer webservers to generate the 3D structural model of Seq15. The names and numbers of RNA nucleotides are labeled in the Figure S1. This model shows that the single-stranded RNA folds into a complex and particular shape. This shape of the single-stranded RNA is critical to the binding interaction between the aptamer and its target. Because shape complementarity, polar contacts, hydrogen bonding interactions, and charge-charge interactions are important for aptamer-target recognition. Table S1: Nucleotides of three RNA aptamers involved in the binding interface with Ang2. Nucleotides at position 10 to 16 and 33 to 99 were marked with bold, underlined text. Table S2: The simulation results of 189 mutant RNA aptamer sequences. The mutated positions in the sequences and the ZRNAK scores of three selected aptamers were marked with bold letters. [file 658712.f1.docx]

**SUPPLEMENTARY MATERIAL**

**Computational selection of RNA aptamer against angiopoietin-2 and experimental evaluation**

Wen-Pin Hu^a,b*^, Jangam Vikram Kumar^a*^, Chun-Jen Huang^c^, Wen-Yih Chen^d^

^a^ Department of Biomedical Informatics, Asia University, Taichung City, Taiwan

^b^ Department of Medical Laboratory Science and Biotechnology, China Medical University, Taichung City, Taiwan

^c^ Graduate Institute of Biomedical Engineering, National Central University, Jhong-Li City, Taiwan

^d^ Department of Chemical and Materials Engineering, National Central University, Jhong-Li City, Taiwan

*Corresponding author: Wen-Pin Hu; Jangam Vikram Kumar

Department of Biomedical Informatics, Asia University

500, Lioufeng Rd., Wufeng, Taichung 41354, Taiwan

TEL: +886-4-23323456 ext.20022; FAX: +886-4-23305737

E-mail: wenpinhu@asia.edu.tw (W.P. Hu); vikramkumar.bioinf@gmail.com (J.V. Kumar)

1. **Materials and methods**

## Computational assay

**
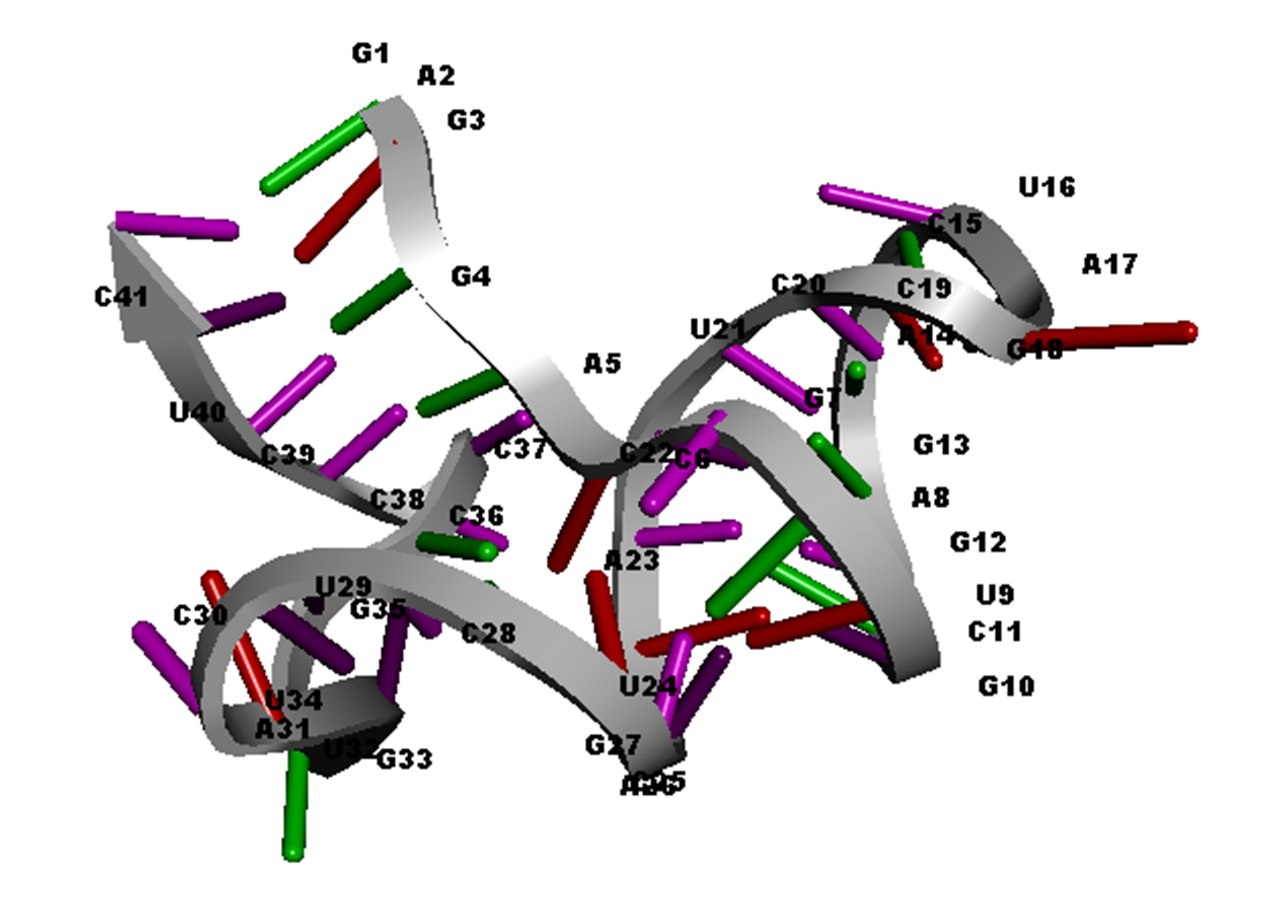
**

**Figure S1.**Model of the 3D Structure of Seq15. This model is generated by using the CentroidFold and RNAComposer webservers.

## The library of mutated RNA sequences

**Table S1.**Nucleotides of three RNA aptamers involved in the binding interface with Ang2. Nucleotides at position 10 to 16 and 33 to 99 were marked with bold, underlined text.

| **Name** | **Nucleotides involved in binding interface with Ang2** |
| --- | --- |
| **Seq1** | A4 G5 C6 C7 U8 C9 **A10 A13 G14 C15 U16** C17 A18 G30 C31 C32 **U33 G34 G35 A36** |
| **Seq2** | U2 A3 A4 C5 C6 A7 U8 C9 **A10 G11 C12 U13 C14 A15 U16A39** C40 |
| **Seq3** | C28 U29 C30 A31 C32 **C33 C34 A35 C36 A37 C38 C39** G40 |
| **Seq4** | C1 C2 A3 C4 C5 G6 A7 U8 C9 **G10 C11 A12 U13 C14 A15 G16** |
| **Seq5** | U9 **C10 U11** G18 C19 C20 G21 A22 U23 C24 A25 U26 C27 G29 C30 G31 C32 **U33** |
| **Seq6** | A5 C6 C7 A8 C9 **G10 C11 U14 A15 U16** C17 A18 G19 C20 U21 A22 A23 **C34 U35 A36** |
| **Seq7** | C6 C7 A8 G9 **U10 C11 A12 C13 C14 A15 U16** |
| **Seq8** | C4 C5 A6 A7 G8 **C10 U11 C12 A13 C14 G15 U16** U17 |
| **Seq9** | G1 G2 A3 G4 C5 G6 C7 A8 A9 G25 A26 A27 C28 U29 C30 C31 G32 |
| **Seq10** | **C13 U14 U15 A16** G29 A30 A31 C32 **U33 C34 C35 A36 G37 A38** |

**Table S1.** (*Continued*) Nucleotides of three RNA aptamers involved in binding interface with Ang2. Nucleotides at position 10 to 16 and 33 to 99 were marked with bold, underlined text.

| **Seq11** | A26 A27 C28 U29 C30 C31 A32 **U33 G34 C35 A36** |
| --- | --- |
| **Seq12** | G23 U24 U25 G26 G28 U29 C30 U31 C32 **G33 U34 C35 G36 A37 A38** |
| **Seq13** | C1 A2 C3 U4 C5 A6 G7 C8 G9 **C10 C11 C12 U13 G14 C15 G16** A17 A18 C32 **C39** U40 |
| **Seq14** | G22 A23 A24 C25 U26 C27 C28 U29 G30 C32 **C33 C34 U35 C36 U37 A38 C39** |
| **Seq15** | **G10 C11 G12 G13 A14** C20 U21 C22 A23 U24 **C37 C38** |
| **Seq16** | **C13 C14 A15 C16** U17 C20 C21 A22 A23 C24 C25 U27 |

## SPR imaging apparatus

The SPR platform is a self- referencing SPR imaging sensor with polarization contrast, which is able to detect the smallest signal corresponding to the change in the refractive index unit (RIU) better than10^-6^ within the operation range of 0.011 RIU. The p-polarized narrow-band light beam (central emission wavelength is 750 nm) impinges on a glass substrate and excites SPs at the metal-dielectric interface. It also can be illustrated in terms of protein surface coverage that the sensor can detect the changes that are as small as 0.2 pg/mm^2^. The SPRi can be utilized by conducting traditional measurements or performing a high-throughput analysis with the use of an array-formatted chip. The SPR chips were prepared by using clean BK7 glass substrates. The glass substrates were pre-coated with an adhesion layer of chromium (thickness approx. 2 nm), and an active gold layer with a thickness of 48 nm was subsequently coated via an evaporation deposition process at pressures below 1×10^-6^ Torr. All experiments were performed at a constant temperature of 25°C and at a flow rate of 50 μl/min. Six flow channels were used in the SPR experiments, and one channel was used as a reference channel.

1. **Results and discussion**

## **2.1.** Selection of aptamers from computational results

**Table S2.**The simulation results of 189 mutant RNA aptamer sequences. The mutated positions in the sequences and the ZRNAK scores of three selected aptamers were marked with bold letters.

| **Type** | **Sequence(**5′-3′**)** | **ZRANK Score** |
| --- | --- | --- |
| **Mutant sequence of Seq1** | ACUAGCCUC**U**UCAGCUCAUGUGCCCCUCCGCC**A**GGAUCAC | -66.174 |
|  | ACUAGCCUC**U**UCAGCUCAUGUGCCCCUCCGCC**G**GGAUCAC | -73.649 |
|  | ACUAGCCUC**U**UCAGCUCAUGUGCCCCUCCGCC**C**GGAUCAC | -69.671 |
|  | ACUAGCCUC**G**UCAGCUCAUGUGCCCCUCCGCC**A**GGAUCAC | -63.625 |
|  | ACUAGCCUC**G**UCAGCUCAUGUGCCCCUCCGCC**G**GGAUCAC | -61.696 |
|  | ACUAGCCUC**G**UCAGCUCAUGUGCCCCUCCGCC**C**GGAUCAC | -71.018 |
|  | ACUAGCCUC**C**UCAGCUCAUGUGCCCCUCCGCC**A**GGAUCAC | -74.2 |
|  | ACUAGCCUC**C**UCAGCUCAUGUGCCCCUCCGCC**G**GGAUCAC | -62.316 |
|  | ACUAGCCUC**C**UCAGCUCAUGUGCCCCUCCGCC**C**GGAUCAC | -66.705 |
|  | ACUAGCCUCA**A**CAGCUCAUGUGCCCCUCCGCCU**A**GAUCAC | -73.514 |
|  | ACUAGCCUCA**A**CAGCUCAUGUGCCCCUCCGCCU**C**GAUCAC | -74.701 |
|  | ACUAGCCUCA**A**CAGCUCAUGUGCCCCUCCGCCU**U**GAUCAC | -87.666 |
|  | ACUAGCCUCA**G**CAGCUCAUGUGCCCCUCCGCCU**A**GAUCAC | -66.493 |
|  | ACUAGCCUCA**G**CAGCUCAUGUGCCCCUCCGCCU**C**GAUCAC | -66.839 |
|  | ACUAGCCUCA**G**CAGCUCAUGUGCCCCUCCGCCU**U**GAUCAC | -61.193 |
|  | ACUAGCCUCA**C**CAGCUCAUGUGCCCCUCCGCCU**A**GAUCAC | -87.829 |
|  | ACUAGCCUCA**C**CAGCUCAUGUGCCCCUCCGCCU**C**GAUCAC | -74.337 |
|  | ACUAGCCUCA**C**CAGCUCAUGUGCCCCUCCGCCU**U**GAUCAC | -65.241 |
|  | ACUAGCCUCAU**A**AGCUCAUGUGCCCCUCCGCCUG**A**AUCAC | -71.17 |
|  | ACUAGCCUCAU**A**AGCUCAUGUGCCCCUCCGCCUG**C**AUCAC | -76.63 |
|  | ACUAGCCUCAU**A**AGCUCAUGUGCCCCUCCGCCUG**U**AUCAC | -68.573 |
|  | ACUAGCCUCAU**G**AGCUCAUGUGCCCCUCCGCCUG**A**AUCAC | -74.78 |
|  | ACUAGCCUCAU**G**AGCUCAUGUGCCCCUCCGCCUG**C**AUCAC | -72.671 |
|  | ACUAGCCUCAU**G**AGCUCAUGUGCCCCUCCGCCUG**U**AUCAC | -73.072 |
|  | ACUAGCCUCAU**U**AGCUCAUGUGCCCCUCCGCCUG**A**AUCAC | -68.47 |
|  | ACUAGCCUCAU**U**AGCUCAUGUGCCCCUCCGCCUG**C**AUCAC | -83.747 |
|  | ACUAGCCUCAU**U**AGCUCAUGUGCCCCUCCGCCUG**U**AUCAC | -64.297 |
|  | ACUAGCCUCAUC**U**GCUCAUGUGCCCCUCCGCCUGG**C**UCAC | -77.279 |
|  | ACUAGCCUCAUC**U**GCUCAUGUGCCCCUCCGCCUGG**G**UCAC | -70.501 |
|  | ACUAGCCUCAUC**U**GCUCAUGUGCCCCUCCGCCUGG**U**UCAC | -73.393 |

**Table S2.**(*Continued*) The simulation results of 189 mutant RNA aptamer sequences. The mutated positions in the sequences and the ZRNAK scores of three selected aptamers were marked with bold letters.

| **Type** | **Sequence(**5′-3′**)** | **ZRANK Score** |
| --- | --- | --- |
| **Mutant sequence of Seq1** | ACUAGCCUCAUC**G**GCUCAUGUGCCCCUCCGCCUGG**C**UCAC | -69.012 |
|  | ACUAGCCUCAUC**G**GCUCAUGUGCCCCUCCGCCUGG**G**UCAC | -60.148 |
|  | ACUAGCCUCAUC**G**GCUCAUGUGCCCCUCCGCCUGG**U**UCAC | -71.464 |
|  | ACUAGCCUCAUC**C**GCUCAUGUGCCCCUCCGCCUGG**C**UCAC | -79.954 |
|  | ACUAGCCUCAUC**C**GCUCAUGUGCCCCUCCGCCUGG**G**UCAC | -76.995 |
|  | ACUAGCCUCAUC**C**GCUCAUGUGCCCCUCCGCCUGG**U**UCAC | -78.571 |
|  | ACUAGCCUCAUCA**A**CUCAUGUGCCCCUCCGCCUGGA**A**CAC | -77.097 |
|  | ACUAGCCUCAUCA**A**CUCAUGUGCCCCUCCGCCUGGA**C**CAC | -67.97 |
|  | ACUAGCCUCAUCA**A**CUCAUGUGCCCCUCCGCCUGGA**G**CAC | -74.329 |
|  | ACUAGCCUCAUCA**C**CUCAUGUGCCCCUCCGCCUGGA**A**CAC | -65.604 |
|  | ACUAGCCUCAUCA**C**CUCAUGUGCCCCUCCGCCUGGA**C**CAC | -70.131 |
|  | ACUAGCCUCAUCA**C**CUCAUGUGCCCCUCCGCCUGGA**G**CAC | -73.143 |
|  | ACUAGCCUCAUCA**U**CUCAUGUGCCCCUCCGCCUGGA**A**CAC | -72.094 |
|  | ACUAGCCUCAUCA**U**CUCAUGUGCCCCUCCGCCUGGA**C**CAC | -66.34 |
|  | ACUAGCCUCAUCA**U**CUCAUGUGCCCCUCCGCCUGGA**G**CAC | -78.013 |
|  | ACUAGCCUCAUCAG**A**UCAUGUGCCCCUCCGCCUGGAU**A**AC | -69.839 |
|  | ACUAGCCUCAUCAG**A**UCAUGUGCCCCUCCGCCUGGAU**G**AC | -67.988 |
|  | ACUAGCCUCAUCAG**A**UCAUGUGCCCCUCCGCCUGGAU**U**AC | -78.518 |
|  | ACUAGCCUCAUCAG**G**UCAUGUGCCCCUCCGCCUGGAU**A**AC | -66.59 |
|  | ACUAGCCUCAUCAG**G**UCAUGUGCCCCUCCGCCUGGAU**G**AC | -68.432 |
|  | ACUAGCCUCAUCAG**G**UCAUGUGCCCCUCCGCCUGGAU**U**AC | -77.178 |
|  | ACUAGCCUCAUCAG**U**UCAUGUGCCCCUCCGCCUGGAU**A**AC | -66.492 |
|  | ACUAGCCUCAUCAG**U**UCAUGUGCCCCUCCGCCUGGAU**G**AC | -61.954 |
|  | ACUAGCCUCAUCAG**U**UCAUGUGCCCCUCCGCCUGGAU**U**AC | -66.717 |
|  | ACUAGCCUCAUCAGC**A**CAUGUGCCCCUCCGCCUGGAUC**C**C | -84.072 |
|  | ACUAGCCUCAUCAGC**A**CAUGUGCCCCUCCGCCUGGAUC**G**C | -67.44 |
|  | ACUAGCCUCAUCAGC**A**CAUGUGCCCCUCCGCCUGGAUC**U**C | -67.844 |
|  | ACUAGCCUCAUCAGC**G**CAUGUGCCCCUCCGCCUGGAUC**C**C | -68.77 |
|  | ACUAGCCUCAUCAGC**G**CAUGUGCCCCUCCGCCUGGAUC**G**C | -64.424 |
|  | ACUAGCCUCAUCAGC**G**CAUGUGCCCCUCCGCCUGGAUC**U**C | -65.86 |
|  | ACUAGCCUCAUCAGC**C**CAUGUGCCCCUCCGCCUGGAUC**C**C | -65.771 |
|  | ACUAGCCUCAUCAGC**C**CAUGUGCCCCUCCGCCUGGAUC**G**C | -77.968 |
|  | ACUAGCCUCAUCAGC**C**CAUGUGCCCCUCCGCCUGGAUC**U**C | -74.137 |

**Table S2.**(*Continued*) The simulation results of 189 mutant RNA aptamer sequences. The mutated positions in the sequences and the ZRNAK scores of three selected aptamers were marked with bold letters.

| **Type** | **Sequence(**5′-3′**)** | **ZRANK Score** |
| --- | --- | --- |
| **Mutant sequence of Seq2** | UUAACCAUC**U**GCUCAUGGCCCCUGCCCUCUCA**U**GGACCAC | -87.021 |
|  | UUAACCAUC**U**GCUCAUGGCCCCUGCCCUCUCA**G**GGACCAC | -78.846 |
|  | UUAACCAUC**U**GCUCAUGGCCCCUGCCCUCUCA**C**GGACCAC | -73.147 |
|  | UUAACCAUC**G**GCUCAUGGCCCCUGCCCUCUCA**U**GGACCAC | -61.103 |
|  | UUAACCAUC**G**GCUCAUGGCCCCUGCCCUCUCA**G**GGACCAC | -71.789 |
|  | UUAACCAUC**G**GCUCAUGGCCCCUGCCCUCUCA**C**GGACCAC | -73.519 |
|  | UUAACCAUC**C**GCUCAUGGCCCCUGCCCUCUCA**U**GGACCAC | -68.534 |
|  | UUAACCAUC**C**GCUCAUGGCCCCUGCCCUCUCA**G**GGACCAC | -67.404 |
|  | UUAACCAUC**C**GCUCAUGGCCCCUGCCCUCUCA**C**GGACCAC | -71.113 |
|  | UUAACCAUCA**A**CUCAUGGCCCCUGCCCUCUCAA**A**GACCAC | -83.898 |
|  | UUAACCAUCA**A**CUCAUGGCCCCUGCCCUCUCAA**U**GACCAC | -70.853 |
|  | UUAACCAUCA**A**CUCAUGGCCCCUGCCCUCUCAA**C**GACCAC | -68.273 |
|  | UUAACCAUCA**U**CUCAUGGCCCCUGCCCUCUCAA**A**GACCAC | -87.682 |
|  | UUAACCAUCA**U**CUCAUGGCCCCUGCCCUCUCAA**U**GACCAC | -63.614 |
|  | UUAACCAUCA**U**CUCAUGGCCCCUGCCCUCUCAA**C**GACCAC | -66.266 |
|  | UUAACCAUCA**C**CUCAUGGCCCCUGCCCUCUCAA**A**GACCAC | -72.281 |
|  | UUAACCAUCA**C**CUCAUGGCCCCUGCCCUCUCAA**U**GACCAC | -76.843 |
|  | UUAACCAUCA**C**CUCAUGGCCCCUGCCCUCUCAA**C**GACCAC | -77.119 |
|  | UUAACCAUCAG**A**UCAUGGCCCCUGCCCUCUCAAG**A**ACCAC | -82.028 |
|  | UUAACCAUCAG**A**UCAUGGCCCCUGCCCUCUCAAG**U**ACCAC | -58.122 |
|  | UUAACCAUCAG**A**UCAUGGCCCCUGCCCUCUCAAG**C**ACCAC | **-97.609** |
|  | UUAACCAUCAG**U**UCAUGGCCCCUGCCCUCUCAAG**A**ACCAC | -69.531 |
|  | UUAACCAUCAG**U**UCAUGGCCCCUGCCCUCUCAAG**U**ACCAC | -70.08 |
|  | UUAACCAUCAG**U**UCAUGGCCCCUGCCCUCUCAAG**C**ACCAC | -78.163 |
|  | UUAACCAUCAG**G**UCAUGGCCCCUGCCCUCUCAAG**A**ACCAC | -87.09 |
|  | UUAACCAUCAG**G**UCAUGGCCCCUGCCCUCUCAAG**U**ACCAC | -84.423 |
|  | UUAACCAUCAG**G**UCAUGGCCCCUGCCCUCUCAAG**C**ACCAC | -67.203 |
|  | UUAACCAUCAGC**A**CAUGGCCCCUGCCCUCUCAAGG**U**CCAC | -69.167 |
|  | UUAACCAUCAGC**A**CAUGGCCCCUGCCCUCUCAAGG**G**CCAC | -70.366 |
|  | UUAACCAUCAGC**A**CAUGGCCCCUGCCCUCUCAAGG**C**CCAC | -73.611 |
|  | UUAACCAUCAGC**G**CAUGGCCCCUGCCCUCUCAAGG**U**CCAC | -74.263 |
|  | UUAACCAUCAGC**G**CAUGGCCCCUGCCCUCUCAAGG**G**CCAC | -79.564 |

**Table S2.**(*Continued*) The simulation results of 189 mutant RNA aptamer sequences. The mutated positions in the sequences and the ZRNAK scores of three selected aptamers were marked with bold letters.

| **Type** | **Sequence(**5′-3′**)** | **ZRANK Score** |
| --- | --- | --- |
| **Mutant sequence of Seq2** | UUAACCAUCAGC**G**CAUGGCCCCUGCCCUCUCAAGG**C**CCAC | -79.122 |
|  | UUAACCAUCAGC**C**CAUGGCCCCUGCCCUCUCAAGG**U**CCAC | -69.499 |
|  | UUAACCAUCAGC**C**CAUGGCCCCUGCCCUCUCAAGG**G**CCAC | -74.227 |
|  | UUAACCAUCAGC**C**CAUGGCCCCUGCCCUCUCAAGG**C**CCAC | -68.816 |
|  | UUAACCAUCAGCU**A**AUGGCCCCUGCCCUCUCAAGGA**A**CAC | -68.767 |
|  | UUAACCAUCAGCU**A**AUGGCCCCUGCCCUCUCAAGGA**U**CAC | -77.011 |
|  | UUAACCAUCAGCU**A**AUGGCCCCUGCCCUCUCAAGGA**G**CAC | -64.889 |
|  | UUAACCAUCAGCU**U**AUGGCCCCUGCCCUCUCAAGGA**A**CAC | -79.081 |
|  | UUAACCAUCAGCU**U**AUGGCCCCUGCCCUCUCAAGGA**U**CAC | -84.05 |
|  | UUAACCAUCAGCU**U**AUGGCCCCUGCCCUCUCAAGGA**G**CAC | -71.727 |
|  | UUAACCAUCAGCU**G**AUGGCCCCUGCCCUCUCAAGGA**A**CAC | -77.511 |
|  | UUAACCAUCAGCU**G**AUGGCCCCUGCCCUCUCAAGGA**U**CAC | -67.982 |
|  | UUAACCAUCAGCU**G**AUGGCCCCUGCCCUCUCAAGGA**G**CAC | -72.09 |
|  | UUAACCAUCAGCUC**U**UGGCCCCUGCCCUCUCAAGGAC**U**AC | -69.207 |
|  | UUAACCAUCAGCUC**U**UGGCCCCUGCCCUCUCAAGGAC**G**AC | -72.46 |
|  | UUAACCAUCAGCUC**G**UGGCCCCUGCCCUCUCAAGGAC**A**AC | -70.948 |
|  | UUAACCAUCAGCUC**G**UGGCCCCUGCCCUCUCAAGGAC**U**AC | -72.441 |
|  | UUAACCAUCAGCUC**G**UGGCCCCUGCCCUCUCAAGGAC**G**AC | -67.28 |
|  | UUAACCAUCAGCUC**C**UGGCCCCUGCCCUCUCAAGGAC**A**AC | -67.681 |
|  | UUAACCAUCAGCUC**C**UGGCCCCUGCCCUCUCAAGGAC**U**AC | -66.611 |
|  | UUAACCAUCAGCUC**C**UGGCCCCUGCCCUCUCAAGGAC**G**AC | -78.931 |
|  | UUAACCAUCAGCUCA**A**GGCCCCUGCCCUCUCAAGGACC**U**C | -68.63 |
|  | UUAACCAUCAGCUCA**A**GGCCCCUGCCCUCUCAAGGACC**G**C | -63.629 |
|  | UUAACCAUCAGCUCA**A**GGCCCCUGCCCUCUCAAGGACC**C**C | -66.074 |
|  | UUAACCAUCAGCUCA**G**GGCCCCUGCCCUCUCAAGGACC**U**C | -86.223 |
|  | UUAACCAUCAGCUCA**G**GGCCCCUGCCCUCUCAAGGACC**G**C | -73.089 |
|  | UUAACCAUCAGCUCA**G**GGCCCCUGCCCUCUCAAGGACC**C**C | -67.327 |
|  | UUAACCAUCAGCUCA**C**GGCCCCUGCCCUCUCAAGGACC**U**C | -66.499 |
|  | UUAACCAUCAGCUCA**C**GGCCCCUGCCCUCUCAAGGACC**G**C | -72.857 |
|  | UUAACCAUCAGCUCA**C**GGCCCCUGCCCUCUCAAGGACC**C**C | -60.727 |

**Table S2.**(*Continued*) The simulation results of 189 mutant RNA aptamer sequences. The mutated positions in the sequences and the ZRNAK scores of three selected aptamers were marked with bold letters.

| **Type** | **Sequence(**5′-3′**)** | **ZRANK Score** |
| --- | --- | --- |
| **Mutant sequence of Seq15** | GAGGACGAU**A**CGGACUAGCCUCAUCAGCUCAU**A**UGCCCCUC | -72.04 |
|  | GAGGACGAU**A**CGGACUAGCCUCAUCAGCUCAU**U**UGCCCCUC | -71.647 |
|  | GAGGACGAU**A**CGGACUAGCCUCAUCAGCUCAU**C**UGCCCCUC | -78.706 |
|  | GAGGACGAU**U**CGGACUAGCCUCAUCAGCUCAU**A**UGCCCCUC | -68.452 |
|  | GAGGACGAU**U**CGGACUAGCCUCAUCAGCUCAU**U**UGCCCCUC | -88.897 |
|  | GAGGACGAU**U**CGGACUAGCCUCAUCAGCUCAU**C**UGCCCCUC | -71.47 |
|  | GAGGACGAU**C**CGGACUAGCCUCAUCAGCUCAU**A**UGCCCCUC | -63.921 |
|  | GAGGACGAU**C**CGGACUAGCCUCAUCAGCUCAU**U**UGCCCCUC | -62.649 |
|  | GAGGACGAU**C**CGGACUAGCCUCAUCAGCUCAU**C**UGCCCCUC | -76.687 |
|  | GAGGACGAUG**A**GGACUAGCCUCAUCAGCUCAUG**A**GCCCCUC | -88.326 |
|  | GAGGACGAUG**A**GGACUAGCCUCAUCAGCUCAUG**G**GCCCCUC | -79.479 |
|  | GAGGACGAUG**A**GGACUAGCCUCAUCAGCUCAUG**C**GCCCCUC | -76.345 |
|  | GAGGACGAUG**G**GGACUAGCCUCAUCAGCUCAUG**A**GCCCCUC | -86.981 |
|  | GAGGACGAUG**G**GGACUAGCCUCAUCAGCUCAUG**G**GCCCCUC | -76.127 |
|  | GAGGACGAUG**G**GGACUAGCCUCAUCAGCUCAUG**C**GCCCCUC | -76.427 |
|  | GAGGACGAUG**U**GGACUAGCCUCAUCAGCUCAUG**A**GCCCCUC | -75.317 |
|  | GAGGACGAUG**U**GGACUAGCCUCAUCAGCUCAUG**G**GCCCCUC | -76.279 |
|  | GAGGACGAUG**U**GGACUAGCCUCAUCAGCUCAUG**C**GCCCCUC | -73.258 |
|  | GAGGACGAUGC**A**GACUAGCCUCAUCAGCUCAUGU**A**CCCCUC | -78.506 |
|  | GAGGACGAUGC**A**GACUAGCCUCAUCAGCUCAUGU**U**CCCCUC | -81.902 |
|  | GAGGACGAUGC**A**GACUAGCCUCAUCAGCUCAUGU**C**CCCCUC | -66.779 |
|  | GAGGACGAUGC**U**GACUAGCCUCAUCAGCUCAUGU**A**CCCCUC | -86.916 |
|  | GAGGACGAUGC**U**GACUAGCCUCAUCAGCUCAUGU**U**CCCCUC | -89.858 |
|  | GAGGACGAUGC**U**GACUAGCCUCAUCAGCUCAUGU**C**CCCCUC | -76.02 |
|  | GAGGACGAUGC**C**GACUAGCCUCAUCAGCUCAUGU**A**CCCCUC | -70.982 |
|  | GAGGACGAUGC**C**GACUAGCCUCAUCAGCUCAUGU**U**CCCCUC | -72.649 |
|  | GAGGACGAUGC**C**GACUAGCCUCAUCAGCUCAUGU**C**CCCCUC | **-93.335** |
|  | GAGGACGAUGCG**A**ACUAGCCUCAUCAGCUCAUGUG**A**CCCUC | -81.726 |
|  | GAGGACGAUGCG**A**ACUAGCCUCAUCAGCUCAUGUG**U**CCCUC | -81.845 |
|  | GAGGACGAUGCG**A**ACUAGCCUCAUCAGCUCAUGUG**G**CCCUC | -77.127 |
|  | GAGGACGAUGCG**U**ACUAGCCUCAUCAGCUCAUGUG**A**CCCUC | -79.628 |
|  | GAGGACGAUGCG**U**ACUAGCCUCAUCAGCUCAUGUG**U**CCCUC | -86.623 |
|  | GAGGACGAUGCG**U**ACUAGCCUCAUCAGCUCAUGUG**G**CCCUC | -79.604 |

**Table S2.**(*Continued*) The simulation results of 189 mutant RNA aptamer sequences. The mutated positions in the sequences and the ZRNAK scores of three selected aptamers were marked with bold letters.

| **Type** | **Sequence(**5′-3′**)** | **ZRANK Score** |
| --- | --- | --- |
| **Mutant sequence of Seq15** | GAGGACGAUGCG**C**ACUAGCCUCAUCAGCUCAUGUG**A**CCCUC | -72.09 |
|  | GAGGACGAUGCG**C**ACUAGCCUCAUCAGCUCAUGUG**U**CCCUC | -67.432 |
|  | GAGGACGAUGCG**C**ACUAGCCUCAUCAGCUCAUGUG**G**CCCUC | -87.531 |
|  | GAGGACGAUGCGG**U**CUAGCCUCAUCAGCUCAUGUGC**A**CCUC | -67.705 |
|  | GAGGACGAUGCGG**U**CUAGCCUCAUCAGCUCAUGUGC**U**CCUC | -59.599 |
|  | GAGGACGAUGCGG**U**CUAGCCUCAUCAGCUCAUGUGC**G**CCUC | -66.643 |
|  | GAGGACGAUGCGG**G**CUAGCCUCAUCAGCUCAUGUGC**A**CCUC | -88.951 |
|  | GAGGACGAUGCGG**G**CUAGCCUCAUCAGCUCAUGUGC**U**CCUC | -78.324 |
|  | GAGGACGAUGCGG**G**CUAGCCUCAUCAGCUCAUGUGC**G**CCUC | -87.948 |
|  | GAGGACGAUGCGG**C**CUAGCCUCAUCAGCUCAUGUGC**A**CCUC | -67.698 |
|  | GAGGACGAUGCGG**C**CUAGCCUCAUCAGCUCAUGUGC**U**CCUC | -72.326 |
|  | GAGGACGAUGCGG**C**CUAGCCUCAUCAGCUCAUGUGC**G**CCUC | -74.909 |
|  | GAGGACGAUGCGGA**A**UAGCCUCAUCAGCUCAUGUGCC**A**CUC | -81.962 |
|  | GAGGACGAUGCGGA**A**UAGCCUCAUCAGCUCAUGUGCC**U**CUC | -78.683 |
|  | GAGGACGAUGCGGA**A**UAGCCUCAUCAGCUCAUGUGCC**G**CUC | -74.477 |
|  | GAGGACGAUGCGGA**U**UAGCCUCAUCAGCUCAUGUGCC**A**CUC | -75.669 |
|  | GAGGACGAUGCGGA**U**UAGCCUCAUCAGCUCAUGUGCC**U**CUC | -72.017 |
|  | GAGGACGAUGCGGA**U**UAGCCUCAUCAGCUCAUGUGCC**G**CUC | **-89.904** |
|  | GAGGACGAUGCGGA**G**UAGCCUCAUCAGCUCAUGUGCC**A**CUC | -77.132 |
|  | GAGGACGAUGCGGA**G**UAGCCUCAUCAGCUCAUGUGCC**U**CUC | -75.5 |
|  | GAGGACGAUGCGGA**G**UAGCCUCAUCAGCUCAUGUGCC**G**CUC | -85.028 |
|  | GAGGACGAUGCGGAC**A**AGCCUCAUCAGCUCAUGUGCCC**A**UC | -78.824 |
|  | GAGGACGAUGCGGAC**A**AGCCUCAUCAGCUCAUGUGCCC**U**UC | -67.014 |
|  | GAGGACGAUGCGGAC**A**AGCCUCAUCAGCUCAUGUGCCC**G**UC | -70.113 |
|  | GAGGACGAUGCGGAC**G**AGCCUCAUCAGCUCAUGUGCCC**A**UC | -77.709 |
|  | GAGGACGAUGCGGAC**G**AGCCUCAUCAGCUCAUGUGCCC**U**UC | -85.681 |
|  | GAGGACGAUGCGGAC**G**AGCCUCAUCAGCUCAUGUGCCC**G**UC | -72.912 |
|  | GAGGACGAUGCGGAC**C**AGCCUCAUCAGCUCAUGUGCCC**A**UC | -79.488 |
|  | GAGGACGAUGCGGAC**C**AGCCUCAUCAGCUCAUGUGCCC**U**UC | -76.647 |
|  | GAGGACGAUGCGGAC**C**AGCCUCAUCAGCUCAUGUGCCC**G**UC | -88.449 |
